# The prevalence of Nonsuicidal Self-Injury (NSSI) in a representative sample of the German population

**DOI:** 10.1186/s12888-016-1060-x

**Published:** 2016-10-19

**Authors:** Paul L. Plener, Marc Allroggen, Nestor D. Kapusta, Elmar Brähler, Jörg M. Fegert, Rebecca C. Groschwitz

**Affiliations:** 1 Department of Child and Adolescent Psychiatry and Psychotherapy, University of Ulm, Steinhoevelstr. 5, 89075 Ulm, Germany; 2 Department for Psychoanalysis and Psychotherapy, Medical University of Vienna, Vienna, Austria; 3 Department of Medical Psychology and Medical Sociology, Universität Leipzig, Leipzig, Germany; 4 Department of Psychosomatic Medicine and Psychotherapy, University Medical Center, Johannes Gutenberg Universität Mainz, Mainz, Germany

**Keywords:** Nonsuicidal Self-Injury, NSSI, Germany, Self-injury, Self-harm

## Abstract

**Background:**

Non-suicidal self-injury (NSSI) is a proposed new “condition for further study” in the DSM-5. To date no prevalence data has been available on this diagnostic entity from a representative sample of the general population.

**Methods:**

A representative sample of the German population (*N* = 2509, mean age = 48.8 years, SD = 18.1, female 55.4 %) completed the NSSI section of the German version of the Self-Injurious Thoughts and Behaviors Interview (SITBI-G).

**Results:**

A history of NSSI at least once during lifetime was reported by 3.1 % of all participants, with higher lifetime prevalence rates in younger age groups. DSM-5 NSSI disorder criteria were met by 0.3 %. The most common function of NSSI was automatic negative reinforcement (e.g. to alleviate negative feelings).

**Conclusions:**

To the best of our knowledge, this is the first study reporting rates for the proposed NSSI category in DSM-5 from a representative sample of the general population. In comparison to findings from community samples of adolescents, adults seem to have lower lifetime prevalence rates of NSSI, thus making it necessary to emphasize prevention and treatment efforts in younger age groups.

## Background

Non-suicidal self-injury (NSSI), defined as deliberate destruction of one’s own body tissue without suicidal intent, which is socially unacceptable has been proposed in the section 3 of the Diagnostic and Statistical Manual, 5^th^ edition (DSM-5) as a “condition for further study” [1]. Although research on self-injury dates back to the 1960s (for review see [2]), the concept of NSSI in the DSM-5 has sparked new research by providing a definition that requires a certain frequency of self-injury (on five or more days within the last year), the presence of a certain functionality of this behavior (i.e. relief from a negative feeling or cognitive state) and the absence of suicidal intent and social acceptance of the behavior [1].

Interestingly, most of the studies reporting on self-injury have been conducted in adolescent or young adult samples so far (for review see [3, 4]). A recent review comparing studies providing longitudinal data, found a peak of NSSI prevalence in adolescence and suggested a decrease during young adulthood [5] which corresponds the only major longitudinal study on self-harm [6].

Research on NSSI in adult and general population samples is rare. One of the first studies reporting data from a stratified and random US general population sample (*n* = 927, mean age: 46, SD = 17, range: 18–90), although not based on the current definition of NSSI (i.e. not using a frequency criterion or functionality of NSSI), reported a history of self-mutilation in 4 % of the population within the last six months, and 0.3 % reported to “often engage” in this behavior [7]. Using random-digit digital dialing, Klonsky [8] examined a US general population sample (*n* = 439, mean age: 55.5, SD = 16.6). The lifetime prevalence rate of NSSI was 5.9 % with 0.9 % reporting NSSI within the last year. Of all participants, 2.7 % had injured five or more times during their lifespan [8]. Looking into studies of self-harm (an umbrella term including both NSSI and other self-injuring behaviors regardless of their suicidal intent) offers a comparable picture [3]. In a randomized cross-sectional survey (Second British National Survey of Psychiatric Morbidity, *n* = 8580, age: 16–74), 2.2 % reported a lifetime prevalence of self-harm [9], whereas another wave of this survey in 2007 showed a 4.9 % prevalence rate of self-harm [10]. With regards to age groups, a decline with older age from 12.4 % in the age group 16–24 to 0.5 % in the age group 75 and above was described [10].

In sum, NSSI is a clinically relevant condition with lifetime prevalence rates between 4 and 5.9 % in the general adult population. The differences in prevalence rates are likely due to different definitions of self-harm behaviors and different methods of assessment. In a systematic review of all epidemiological studies, the mean lifetime prevalence of NSSI (after adjustment for methodological factors) was estimated to be 15.4 % in adolescents, 10.5 % in young adults and 4.2 % in adults [4].

Since the definition of NSSI has been included in the DSM-5 only recently, only few studies have used this definition. From a Swedish school sample (*n* = 3060), 7–17 years of age, a rate of 6.7 % was reported for the current NSSI criteria [11]. Higher rates have been reported from clinical samples. In the US, Selby et al. [12] reported a prevalence of 11 % in adult psychiatric outpatients (*n* = 571). In a clinical adolescent sample (*N* = 198, 12–18 years) a NSSI rate of 49.5 % was reported by Glenn & Klonsky [13]. This rate is comparable to findings from German clinical adolescent samples (47.0–49.6 %) [14, 15].

Although most prevalence studies on NSSI stem from the US and Canada [3, 4], in recent years epidemiological research on NSSI has also increasingly originated from Germany, an European country with a high prevalence of NSSI in adolescence [16]. In a first epidemiological study, Brunner et al. [17] reported a prevalence rate of 10.9 % for occasional and 4 % for repetitive deliberate self-harm (including behaviors with suicidal intent) from a German adolescent community sample (*n* = 5759, mean age: 14.9, SD = 0.73). Using an NSSI definition (and therefore excluding behavior with suicidal intent) and comparing community samples from the US and Germany (*n* = 665, mean age: 14.8), Plener et al. [18] described a lifetime prevalence rate of 25.6 %. More recently, a lifetime prevalence rate of 20.7 % was reported from another German adolescent community sample (*n* = 452, age range: 14–17) [19]. Comparing German speaking school samples from Austria, Germany and Switzerland (n = 1339, mean age: 14.99, SD = 0.79), 6-month prevalence rates of NSSI were highest in German students (14 % vs. 11 % in Austria and 7.6 % in Switzerland) [20]. In a recent European comparison study (*n* = 12086, mean age: 14.9, SD = 0.89) of deliberate self-injury (a definition including NSSI but not explicitly excluding suicidal behavior) a lifetime prevalence rate of 35.1 % was reported from Germany, (repetitive: 12.6 %, occasional: 22.9 %) thus setting Germany second only to France among the 11 participating nations [16]. Despite these multiple studies from adolescent samples, only one study so far reported prevalence rates of NSSI from a young adult sample from Germany. In a study of 714 medical students (mean age: 23.1, age range: 18-35 years) a lifetime prevalence of 14.3 % of NSSI was found, thus showing lower rates than in all community samples of adolescents, that have been researched in Germany up to that point [21].

So far, no data on the current DSM-5 definition of NSSI in the general German population has been available. We sought to address this gap by conducting a prevalence study in a representative sample of the German population and examining the association of NSSI with sociodemographic and economic factors.

## Methods

### Study sample

A representative sample comprising the general population of Germany (regarding age, sex, region of residence, and education) was selected by a demographic consulting company (USUMA, Berlin, Germany) in 2014. Members of households meeting inclusion criteria (minimum age 14 years, sufficient knowledge of German language) were randomly selected (Kish selection grid). Participants were visited by a study assistant in person. If not at home, a maximum of three attempts was made to reach the respondent. The assistant informed about the study, obtained written informed consent of the participants and waited until the participant had completed the self-report questionnaires. Subjects were able to ask the research assistant for help, if they had not understood the meaning of a question. A total of 2513 subjects agreed to participate and completed the questionnaires. However, four of those participants did not provide answer to the question whether they had self-injured. Those participants were excluded from analyses. Therefore, a total of 2509 participants were included. The average age of participants was M = 48.8 years (SD = 18.1; range 14 to 94 years), with 55.4 % being female (see Table 1). Detailed characteristics of the sample are given in Table 1. The survey was in concordance with the Declaration of Helsinki, met ethical guidelines of the international code of Marketing and Social Research practice by the International Chamber of Commerce and the European Society for Opinion and Marketing Research and was approved by the Institutional Review Board (IRB) of the University of Leipzig.

**Table 1:** Socio-demographic data of participants, Total *N* = 2509

Socio-demographic data	M (SD)	range
Age (*N* = 2509)	48.8 (18.1)	14–94 years
	*N*	%
Gender (*N* = 2509)	1391 female	55.4 female
Education (*N* = 2440) - Student - No graduation - ‘Hauptschule’ - ‘Realschule’/’POS’ - ‘Gymnasium’ - University degree	74 59 788 1000 290 229	3.0 2.4 32.3 41.0 11.9 9.4
Currently unemployed (*N* = 2509)	136	5.4
Living with partner (*N* = 2493)	1373	55.1
Place of living (urban vs. rural) (*N* = 2509)	2183	87.0
Ever been in psychotherapeutic treatment (*N* = 2495)	443	17.8

### Assessment of non-suicidal self-injury

Non-suicidal self-injury was assessed using the NSSI module from the German version of the Self-Injurious Thoughts and Behaviors Interview (SITBI-G). The original SITBI has been shown to reliably assess NSSI as well as suicidality, with strong interrater reliability (k = .99, r = 1.0), test-retest reliability (k = .70) and concurrent validity (k = .87) (Nock et al., 2007). The German translation (SITBI-G) also showed moderate to good test-retest reliability (k = .60), a very good interrater reliability (k = 1.0), and a good construct validity (k = .89) [22]. The SITBI-G has been shown to be a reliable and valid instrument to assess Non-Suicidal Self-Injury disorder, as suggested in the DSM-5, section 3 [22]. The questions from the SITBI-G were changed to self-report using paper and pencil format, thus not administered as interview, a procedure that has been used in a comparison study of assessment instruments before, showing very good psychometric properties (reliability: K = 1.0, external validity using the Functional Assessment of Self-Mutilation: K = 1.0, r = .99) [23]. Details about lifetime as well as 12-month frequencies of NSSI were assessed. Furthermore, age of onset and offset, as well as methods and functions of NSSI were evaluated. NSSI is defined in the SITBI as “hurting yourself without wanting to die (for example, cutting or burning)” [24]. The question for lifetime prevalence of NSSI as: “Have you ever actually engaged in NSSI?”, then leading on to more detailed questions if answered positively (i.e. lifetime- and 12-months frequencies, methods, functions). We furthermore asked if participants have received treatment or had mental health problems (lifetime and recently), or have been taking psychotropic medication (lifetime and recently). Results are shown both for participants reporting at least one incident of NSSI during their lifetime and for those who currently fulfill diagnostic criteria of DSM-5 NSSI disorder.

Participants rated functions of their self-injurious behaviors, based on the four factor model of [25], on a scale from 0 to 4 with regards to automatic negative reinforcement (ANR, “to get rid of negative feelings”), automatic positive reinforcement (APR, “to feel something”), social negative reinforcement (SNR, “to get away from others/out of doing something”), and social positive reinforcement (SPR, “to communicate with others/get attention”). Each function was assessed by one question (as stated above).

### Statistical analyses

IBM SPSS Statistics, Version 20.0 was used to conduct statistical analyses. For analyzing significant differences between groups (i.e. NSSI and no NSSI), *t*-tests were performed. Chi^2^-tests were used to test for differences in dichotomous variables between groups. Effect sizes for all significant measures were calculated (Cohen’s d for *t*-tests, and phi for Chi^2^-test). Significance was set at *p* < 0.05.

## Results

### Prevalence of NSSI

Out of 2509 participants, 78 (3.1 %) stated that they have had engaged in NSSI at least once in their life, with decreasing rates in higher age categories (see Table 2). Women reported a significantly higher rate of NSSI (4.1 % vs 1.9 %, Chi^2^ = 10.136, *p* = 0.001) than men. The mean age of NSSI onset was 17.25 years (range: 8–54 years; SD = 8.9), the mean age at which NSSI was stopped was 26.74 years (range: 12–55 years; SD = 12.9). On average, participants had engaged in NSSI for 9.3 years (SD = 11.2, min = < 1 year, max = 41 years).

**Table 2:** Comparison between participants with or without a history of NSSI

	NSSI lifetime (%)	No NSSI (%)	Total (%)
Total (*N* = 2509)	78 (3.1)	2431 (96.9)	2509 (100)
Gender (female) (*N* = 2509)	57 (73.1)	1334 (54.9)	1391 (55.4)
Age group (*N* = 2509)			
Age 14–24	21 (26.9)	256 (10.5)	277 (11)
Age 25–34	22 (28.2)	352 (14.5)	374 (13.8)
Age 35–44	14 (17.9)	359 (14.8)	373 (14.9)
Age 45–54	8 (10.3)	463 (19.0)	471 (18.8)
Age 55–64	10 (12.8)	451 (18.6)	461 (18.4)
Age 65–74	1 (1.3)	346 (14.2)	347 (13.8)
Age 75 >	2 (2.6)	204 (8.4)	206 (8.2)
Place of living (*N* = 2509)			
Urban place of living	66 (84.6)	2117 (87.1)	2183 (87.0)
Rural place of living	12 (15.4)	314 (12.9)	326 (13.0)
Employment (*N* = 1505)			
Full time	18 (36.0)	976 (40.1)	994 (66.0)
Part time: 15–34 h	12 (24.0)	284 (11.7)	296 (14.9)
< 15 h	4 (8.0)	75 (3.1)	79 (5.2)
unemployed	16 (32.0)	120 (4.9)	136 (9.0)
Profession (*N* = 2474)			
Never worked	11 (14.5)	148 (6.1)	159 (6.4)
Blue collar	10 (13.2)	285 (11.7)	295 (11.9)
Higher skilled worker	1 (1.3)	318 (13.1)	319 (12.9)
Farmers	0 (0)	14 (.6)	14 (.6)
Free employed (e.g physicians)	1 (1.3)	36 (1.5)	37 (1.5)
Employee	49 (64.5)	1389 (57.1)	1438 (58.1)
Civil servants	1 (1.3)	79 (3.2)	80 (3.2)
Self employed	3 (3.9)	129 (5.3)	132 (5.3)
Household income (*N* = 2416)			
Household income < € 1250	21 (18.0)	406 (16.7)	427 (17.7)
Household income € 1250–2500	35 (46.7)	1032 (42.5)	1067 (44.2)
Household income < € 2500	19 (25.3)	903 (37.1)	922 (38.2)
Member of religious community (*N* = 2498)			
Catholic	25 (32.1)	759 (31.2)	784 (31.4)
Protestant	27 (34.6)	854 (35.1)	881 (35.3)
Other Christian, Greek and Russian orthodox	1 (1.3)	30 (1.2)	31 (1.2)
Islamic	1 (1.3)	55 (2.3)	56 (2.2)
Jewish	0 (0)	5 (.2)	5 (.2)
Buddhistic	0 (0)	6 (.2)	6 (.2)
Other	2 (2.6)	40 (1.6)	42 (1.7)
Not involved in religious community	22 (28.2)	671 (27.6)	693 (27.7)
Partnership (*N* = 2493)			
Living with partner	29 (37.7)	1344 (55.3)	1373 (55.1)
Single	48 (62.3)	1072 (44.1)	1120 (44.9)
Citizenship (*N* = 2509)			
German citizenship	76 (97.4)	2347 (96.5)	2423 (96.6)
Citizenship other than German	2 (2.6)	84 (3.5)	86 (3.4)
Ever been in psychological, psychotherapeutic or psychiatric treatment (*N* = 2495)	52 (67.5)	391 (16.1)	443 (17.8)

There was no difference in NSSI prevalence between people living in cities and in the countryside (Chi^2^ = .407, *p* = .523) as well as no difference with regards to being member of a religious group (Chi^2^ = .009, *p* = .926), specific religious group (Chi^2^ = 1.087, *p* = .993), country of citizenship (Chi^2^ = .181, *p* = .670) and region of residence in Germany (Chi^2^ = 19.365, *p* = .250).

Two thirds (66.7 %, f: 68.4 %, m: 65.0 %; Chi^2^ = .079, *p* = .787) of participants with NSSI reported previous treatment due to mental health problems, whereas only 16.2 % of participants without NSSI reported a history of such treatment. Participants reporting a history of NSSI were therefore significantly more likely to have received mental health treatment in the past than people without a history of NSSI (Chi^2^ = 134,810, *p* < .001).

Unemployed participants were more likely to report a history of NSSI (Chi^2^ = 32.631, *p* < .001), as were people with a lower household income (Chi^2^ = 8.118, *p* = .017). In addition, people who did not live with a partner showed a higher prevalence rate of NSSI (Chi^2^ = 9.736, *p* = .002).

In the whole sample, only seven people (0.3 %) met criteria for NSSI disorder as proposed in section three of the DSM-5, with all of them being female (see Table 3).

**Table 3:** DSM-5 NSSI disorder and age groups (*N* = 2509)

	DSM-5 NSSI disorder (%)	No DSM-5 NSSI disorder (%)
Total	7 (0.3)	2502 (99.7)
Age 14–24	4 (1.4)	273 (98.6)
Age 25–34	1 (0.3)	373 (99.7)
Age 35–44	1 (0.3)	373 (99.7)
Age 45–54	0 (0)	471 (100)
Age 55–64	1 (0.2)	460 (99.8)
Age 65–74	0 (0)	347 (100)
Age 75 >	0 (0)	206 (100)

### Functions of NSSI

On average, automatic negative reinforcement (ANR) was rated the highest, with M = 2.25 (SD = 1.5), followed by automatic positive reinforcement (APR) (M = 1.79, SD = 1.5), and social negative reinforcement (SNR) (M = 1.64, SD = 1.5) as well as social positive reinforcement (SPR) (M = 1.64, SD = 1.4). Participants who met DSM-5 criteria for NSSI reported significantly higher scores then those with subthreshold NSSI only for APR (*t* = 3.2, *p* = .002) but not for other functionalities (see Table 4).

**Table 4:** Functions of NSSI in participants with NSSI fulfilling or not fulfilling criteria for DSM-5 NSSI disorder

	M_NSSI DSM-5_ (SD)	M_NSSI_noDSM-5_ (SD)	df	T	p
Automatic negative reinforcement	2.86 (.9)	2.19 (1.5)	74	1.16	.25
Automatic positive reinforcement	3.43 (1.1)	1.62 (1.4)	74	3.22	.002
Social negative reinforcement	2.14 (1.8)	1.59 (1.4)	74	.94	.35
Social positive reinforcement	1.29 (1.3)	1.68 (1.5)	74	-.70	.49

The frequency of NSSI (lifetime) was positively correlated with higher ratings of ANR (*r* = .30, *p* = .008) and APR (*r* = .26, *p* = .023) but was not associated with social functions of NSSI.

## Discussion

We conducted a study on the prevalence of NSSI in a representative sample of the German population. Out of 2509 participants, a lifetime history of NSSI was reported in 3.1 %, with 0.3 % meeting the diagnostic criteria of NSSI disorder according to DSM-5. The rate of 3.1 % is somewhat lower as compared to recent epidemiological studies from the US (5.9 %) or from the UK (4.9 %) [8, 10]. However, it is comparable to a recent waiting room study of 1171 patients of general practitioners (mean age: 52.9, SD: 17.0) in Northern Italy a 2.2 % prevalence rate of NSSI was described [26].

When comparing studies conducted in different age groups in Germany, adults show a lower lifetime prevalence than adolescents [16, 18–20] or young adults [21]. This seems to be counterintuitive since lifetime prevalence should increase over the life span, as risks and exposure time accumulate. There are two possible reasons to explain this finding: First, rates of NSSI have increased within recent years, second, adults report lower rates due to recall bias or due to re-attribution [27]. Whereas an increase in rates of NSSI has been discussed e.g. in the lay press (e.g. [28]) and has been shown between 2002 and 2007 in a British national survey for self-harm [10], two systematic reviews have not found signs of increasing rates within a 10 year period after methodological factors were controlled for [3, 4]. Furthermore, a study presenting a five year follow-up on cohorts of the same age groups also failed to show an increase of prevalence rates over time [29]. However, this could also be due to the fact that epidemiological NSSI research in itself is a rather young scientific field, only dating back to 1998 with regards to the first epidemiological study from the general population [7]. It may well be that NSSI was not a prevalent phenomenon when the majority of participants of our study were in their teenage years, but has increased in the years before the first prevalence studies in adolescents [30] were conducted and has reached a plateau and stayed stable for the last couple of years. To examine possible time trends in the prevalence in NSSI, our results should be replicated in future with the same instruments and methods. The other explanation for the higher prevalence of NSSI in adolescents, is a possible recall bias, a well-known phenomenon in research relying on retrospective data [31]. The phenomenon of underreporting in adulthood in comparison to adolescence has been described for suicidal behavior as well [32] and could be due either to inaccurate memory or a re-interpretation of former adolescent behavior as not relevant or serious enough to report. Given that research in NSSI is young, and only one longitudinal study with a follow-up of 15 years is so far available for deliberate self-harm [6], only monitoring of trends building on the recent studies of NSSI in comparable samples throughout the next years to come will have the potential to answer the question whether NSSI is increasing or not. Our findings support the notion that NSSI can be perceived as a considerable phenomenon in adolescence but is rather rarely present in adulthood. It remains unclear why adolescents stop to self-injure when growing older, as suggested by several longitudinal studies [5]. As NSSI is often used as a coping strategy for aversive emotional states (which could be shown in our results as well), it may decrease as soon as the ability for emotional regulation increases over the years and new coping strategies are acquired to regulate psychological or mental distress, both of which are risk factors for NSSI [33, 34].

With regards to the motivations for NSSI, most participants with a history of NSSI reported that ANR (e.g. self-injury to alleviate negative affect) served as their main function, with significantly higher ratings in those participants fulfilling DSM-5 NSSI disorder criteria. Furthermore, the function of ANR was significantly associated with the frequency of NSSI. Interestingly, although not statistically significant, participants fulfilling DSM-5 NSSI disorder criteria reported lower levels with regards to positive social reinforcement (e.g. receiving help from others after NSSI) as motivation for NSSI. The finding of ANR as main motivation for NSSI is in line with literally every study that has been published on functions of NSSI (e.g. [24, 25, 35–37], for review: [38]). The positive association of an ANR function with frequency of NSSI has been reported by Klonsky [37] as well. In a recent latent class analysis of adolescents with NSSI, the classes with the highest frequency of NSSI also reported more intrapersonal functions [39]. The higher ratings of the ANR function in the DSM-5 NSSI disorder group may point to the fact that this group has an especially strong benefit from regulating emotions through NSSI, which could be hypothesized to be linked to a stronger neurobiological association (for review see [40]).

The mean starting age for NSSI was described to peak around 17 years of age. This onset age seems older than findings shown in other German adolescent samples, which reported an onset of NSSI around age of 13 [18]. This result could be due to the fact that the sample was mainly consisting of adults and only rather few adolescent participants. Compared to another sample of adults, the starting age is in line with an age of 16, reported from an US general population sample [8] or 15.2, reported from a Canadian general population sample of adolescents and young adults [41]. The finding of higher rates of NSSI in people who are unemployed and in people who report a lower household income is in line with the findings by Young et al. [42].

The rate of 66.7 % of those with a history of NSSI, who have received any form of psychological therapy is slightly higher than in a study of the general British population, where 35 % of men and 47 % of women with a history of self-harm reported receiving psychological help for their problems [10]. The recipients of treatment in our study were equally distributed among genders.

Limitations: Several limitations apply when interpreting the results of this study. First, our data rely on retrospective self-report, thus being susceptible to memory bias. It has been described that studies on NSSI conducted by self-rating questionnaires yield higher prevalence rates than interview studies [4], as anonymity offers a higher chance for “true” answers. In our study procedure, the research assistant handed over and collected an envelope containing the questionnaire, but did not interfere with completing the questionnaire without being asked. Therefore, the possibility to answer truly but also being able to explain if questions arose was given, thus diminishing the potential for misunderstandings and hence inaccurate answers. Second, the SITBI and SITBI-G up to now has only been used in adolescent samples. Although this is the first use in an adult sample, we have no reason to believe that the validity of the SITBI could be compromised in adults. Furthermore, the SITBI-G was first used in a paper- and pencil version in this study and no psychometric properties were assessed. However, since psychometric properties of the German and English version of the SITBI (as an interview) were very comparable to each other [22] and a paper- and pencil version of the English version showed very good psychometric properties, it is very likely that the German paper- and pencil version would have yielded similar results. Nevertheless, future research evaluating psychometric properties of the paper- and pencil version would be needed in order to verify this statement.

## Conclusions

In conclusion, this study adds to the small amount of literature available on rates of NSSI in representative samples of the general population by introducing the prevalence rates of the recently proposed NSSI disorder in NSSI. The findings of our study point to the fact that while NSSI is widely distributed in the general adolescent population, fewer adults report a history of NSSI. This finding could inform preventive and treatment interventions, which should be tailored towards the need of an adolescent population first. Furthermore, specific treatments should focus on emotion regulation, since the reduction of negative emotions was rated highly in all participants with NSSI, and especially in those with more frequent NSSI.
